# Non-verbal Adaptation to the Interlocutors' Inner Characteristics: Relevance, Challenges, and Future Directions

**DOI:** 10.3389/fpsyg.2021.612664

**Published:** 2021-04-20

**Authors:** Valerie Carrard

**Affiliations:** ^1^Swiss Paraplegic Research (SPF), Nottwil, Switzerland; ^2^Department of Health Sciences and Medicine, University of Lucerne, Lucerne, Switzerland

**Keywords:** behavioral adaptability, non-verbal behavior, expectations, preferences, social interaction

## Abstract

Human diversity cannot be denied. In our everyday social interactions, we constantly experience the fact that each individual is a unique combination of characteristics with specific cultural norms, roles, personality, and mood. Efficient social interaction thus requires an adaptation of communication behaviors to each specific interlocutor that one encounters. This is especially true for non-verbal communication that is more unconscious and automatic than verbal communication. Consequently, non-verbal communication needs to be understood as a dynamic and adaptive process in the theoretical modeling and study of social interactions. This perspective paper presents relevance, challenges, and future directions for the study of non-verbal adaptation in social interactions. It proposes that non-verbal adaptability is more pertinently studied as adaptation to interlocutor's inner characteristics (i.e., expectations or preferences) than to interlocutor's behaviors *per se*, because behaviors are communication messages that individuals interpret in order to understand their interlocutors. The affiliation and control dimensions of the Interpersonal Circumplex Model are proposed as a framework to measure both the interlocutors' inner characteristics (self-reported) and the individuals' non-verbal responses (external coders). These measures can then be compared across different interactions to assess an actual change in behavior tailored to different interlocutors. These recommendations are proposed in the hope of generating more research on the topic of non-verbal adaptability. Indeed, after having gathered the evidence on average effects of non-verbal behaviors, the field can go further than a “one size fits all” approach, by investigating the predictors, moderators, and outcomes of non-verbal adaptation to the interlocutors' inner characteristics.

## Introduction

“*It is not the strongest of the species that survives, not the most intelligent that survives. It is the one that is the most adaptable to change.”*—Charles Darwin

As stated in this famous quote attributed to Darwin, adaptation might be the most important quality for the survival of species, and this could still apply to modern human beings. As humans, we inexorably need to adapt to new situations, roles, and environments, and as social beings, we need to constantly adapt to each interactional partner we encounter. Every social encounter happens in a specific context, bears its specific goals, and involves specific interlocutors (i.e., interactional partner). Each of these elements requires an adaptation of communicative behaviors to achieve successful interactions. Street (1992) thus defines interpersonal communications as “processes of personal and mutual influence that unfold according to the characteristics of the individuals (e.g., attitudes, knowledge, communicative style) and the interactive processes related to how interactants adapt their communication to one another” (p. 1155). Consequently, communication behaviors need to be understood as a dynamic and adaptive process in the theoretical modeling of social interactions, but also in the way it is studied.

Still, many social psychology studies aim at identifying the behaviors that would relate to successful interactions overall, in spite of the interlocutor and situation at hand. Which communication style should physicians display? What is the best leadership behavior? How much should I smile during a job interview? The answer to these questions might be “it depends.” Investigating the impact of communication behaviors from an average perspective is obviously important, because it provides overall guidelines that are more likely to trigger the intended output. However, a “one size fits all” perspective disregards the specificities of each interactional partner. For instance, giving a lot of information and establishing shared decision making might be the medical communication style that is linked to better patient outcomes on average, but some patients actually prefer less information or more passivity (Kiesler and Auerbach, 2006). Thus, a better way to achieve successful interaction outcomes may require promoting flexible adaptation instead of a set of behaviors to apply in every interaction. However, fewer studies focused on the beneficial effect of adapting one's behavior in social interaction. The literature on the subject comes mostly from the communication (Brennan and Hanna, 2009) and the medical interaction fields (Kiesler and Auerbach, 2006) and mainly focuses on the adaptation of verbal behaviors. In comparison, the adaptation of non-verbal behaviors (NVBs) has been scarcely investigated.

NVB is not merely an automatic outward display of inner states; rather, they are unambiguously social signals that are produced with a communicative purpose. Studies have indeed showed that NVBs are produced more intensely in social interactions and are directly linked to social consequences (Schmidt and Cohn, 2001). Furthermore, the establishment of critical interaction styles, such as power, relies largely on non-verbal signals, such as facial expressions, gestures, and spatial management (Hall et al., 2005). Behavioral adaptation of non-verbal signals seems especially critical, because they are more automatically processed in comparison to verbal communication (Choi et al., 2005).

This perspective paper strives to present relevance, challenges, and future directions for the study of non-verbal adaptation in social interactions in terms of mutual adjustment of NVB (and not in the Darwinian's sense of adaptation). First, it will be underlined that individuals do not merely adapt to the interlocutors' behaviors *per se*, but also to the individuals' interpretation of what the behaviors convey about the interlocutors' inner state (i.e., expectations or preferences). Then, the operationalization challenges of non-verbal adaptation to the interlocutor's inner characteristics will be discussed.

## Behavioral Contagion, Behavior-to-Behavior Adaptation, and Adaptation to the Interlocutor's Inner Characteristics

Research on behavioral mutual influences in social interactions first focused on unintentional and automatic NVB contagion such as mimicry (i.e., imitation of speech inflections, facial expressions, and postures; Chartrand and Bargh, 1999) and interactional synchrony (i.e., timely coordination of verbal behvior and NVB; Condon and Ogston, 1967). Later theoretical models such as the Communication Accommodation Theory (Giles et al., 1987, 1991) conceptualize intentional behavior-to-behavior adaptation strategies such as convergence (displaying the same behaviors) and divergence (displaying the opposite behaviors) moves, used to engage or disengage from the interaction. Mimicry, synchrony, and behavior-to-behavior adaptation looking at similarities and dissimilarities between two interactants' behaviors have been extensively studied in different fields such as communication and clinical interactions (Hatfield et al., 2014; Leclère et al., 2014; Soliz and Giles, 2014). However, verbal behaviors or NVBs are not merely oral or visual features. As underlined by several theoretical models such as the Expectancy Violations Theory (Burgoon and Hale, 1988), the Sequential–Functional Model (Patterson, 1982), or the Interaction Adaptation Theory (Burgoon et al., 2007), behaviors are communication tools carrying a message, and the interactants will interpret the meaning of this message and adapt their response accordingly. Evidence supports that individuals adapt their behaviors to the interlocutor's inner characteristics, which are interpreted through displayed behaviors. For instance, research showed that children adapt their communication behaviors (e.g., initiation/response behaviors, gazing, voice frequency, number of words used, and length of vowels) according to their interactional partners' abilities (i.e., hearing or sight impairment) and preferences (wanting help or not; Ganea et al., 2018; Granlund et al., 2018; Gampe et al., 2019). Similarly, surgeons report adapting their guidance in decision-making according to their perception of patients' autonomy, communication competence, interpersonal style, and ability to manage illness (Dekkers et al., 2018). Thus, adaptation to interlocutors' inner characteristics as perceived through behavioral cues is a reality of social interaction.

The interlocutors' inner characteristics that one might make assumptions about and thus adapt to can be conceptualized as expectations (defined by social norms, roles, and the situation at hand) or preferences (defined by personality and emotional state) related to the interaction. For instance, one can interpret an interlocutor's increased interpersonal distance as conveying his or her expectations for a more formal interaction due to a hierarchically defined relationship or his or her preferences for a colder exchange, due to an introverted personality. The multiple behavioral input individuals receive from their interlocutors provide many cues that will add up and enable individuals to determine the behaviors expected or preferred by their interlocutors. In short, most of the adaptation, and especially the more conscious and intentional adjustments, will be based on how behaviors are interpreted, as cues of the interlocutor's inner characteristics. This process of adaptation to the interlocutor's inner characteristics has however been less investigated in comparison to behavior-to-behavior adaptation. This lack of research could be due to the scarcity of extant methodological guidelines available to study it.

## Recommendations for the Investigation of Non-Verbal Adaptation to the Interlocutor's Inner Characteristics

Investigating how individuals adapt their NVB to the inner characteristics of their interlocutors requires three steps of operationalization: (1) assessing the inner characteristics of the interlocutor, (2) assessing the non-verbal response of the individual, and (3) assessing how the NVB is adapted to the interlocutor's inner characteristics. Each of these three steps implies important methodological considerations in terms of collection method, timing of assessment, and operationalization.

Regarding the collection method, many studies rely on self-report. The inner characteristics (i.e., expectations or preferences) of the interlocutor, like any inner world variable, are indeed best measured with self-report. However, using self-reported measures of displayed behaviors is subject to many biases such as social desirability and recall bias (Paulhus and Vazire, 2007). Indeed, even when individuals are conscious of their behaviors, they are inaccurate in reporting them (Jones, 1991). Moreover, the rating of behaviors (one's own or the interactive partner's) is highly influenced by the overall impression of and satisfaction with the interaction (Kiesler and Auerbach, 2006). Thus, an observer coding is a more reliable operationalization of individuals' NVB.

The timing of the assessment is also critical to avoid biases. It is indeed important to note that the interlocutors' inner characteristics are more reliably measured before the interaction of interest, because a post-interaction assessment would be biased by the overall impression of the interaction and interaction outcomes would then be confounded with the measure of interlocutor's expectations or preferences.

Most importantly, the operationalization of interlocutor's inner characteristics and non-verbal answer must be chosen carefully. Assessing how non-verbal response is adapted to the expectations or preferences of an interlocutor implies the comparison of observed behaviors to some inner characteristics. To do so, both need to be assessed with similar operationalization. To this end, researchers can rely on a theoretical framework, which clusters interpersonal behaviors and attitudes according to their functions: the Interpersonal Circumplex Model (ICM; Wiggins and Trobst, 1997). The ICM proposes that two basic dimensions underlie all human interactions: control and affiliation. Control is the dimension pertaining to the verticality of human interaction going from dominance to submission, whereas affiliation represents the horizontality with a continuum from friendliness to hostility. The ICM has served as a theoretical model for many studies of personality (Smith, 1992; Smith et al., 1996) and interpersonal behaviors (Moskowitz et al., 2001; Kiesler and Auerbach, 2003; Newton et al., 2005), because the control and affiliation dimensions can be applied to describe both behavioral display and inner characteristics. Indeed, several validated questionnaires based on the ICM can be used to measure long-standing personality dispositions (e.g., the Interpersonal Checklist; LaForge and Suczek, 1955), interactional preferences (e.g., the Patient-Practitionner Orientation Scale; Krupat et al., 2000), and behaviors (e.g., the Impact Message Inventory or the Interpersonal Transactions-Revised; Kiesler, 1987; Kiesler and Schmidt, 1993). The most versatile instrument measuring the control and affiliation dimensions of the ICM is the Revised Interpersonal Adjective Scale (IAS-R; Wiggins et al., 1988). The IAS-R is composed of 64 adjectives describing interpersonal characteristics mapped on the ICM such as “unsympathetic” and “kind” for the affiliation continuum or “shy” and “assertive” for the control continuum. This scale is versatile, because with a small adaptation of the instructions, researchers can use it to assess expectations or preferences for an upcoming interaction as well as actual interactional behaviors displayed. With the following instruction: “Please indicate the extent to which the following adjectives correspond to your *expectations* regarding the behavior of your interlocutor in the upcoming interaction” or with the same instruction asking about *preferences*, researchers can measure the extent to which interlocutors expect or prefer an affiliative (“unsympathetic” or “kind”) or controlling (e.g., “shy” or “assertive”) interactional partner. These expectations or preferences can then be compared to the extent of affiliation and control the partner actually displayed during the interaction. Note that whether one wants to assess expectations or preferences will depend on the objective of the study and its context. Indeed, in some social interactions such as job interviews, expectations about the interlocutor's communication behavior seems more central than preference, whereas preferences might be more important in other context such as medical interactions, where patient preferences are critical, according to the currently recommended patient-centered approach. In any case, expectations are not synonymous to preferences, and the two might differ considerably. Thus, adaptation to preferences or adaptation to expectations should be measured separately and not aggregated.

The displayed NVB of the interactional partner can also be measured with the IAS-R adjectives as rated by external coders with the following instruction: “Based on the displayed behaviors, indicate the extent to which the following adjectives correspond to the interactional partner.” Research has indeed used the IAS or a shortened version of it with external coders and reported satisfactory reliability and convergent validity (i.e., related to self-reported IAS or observed discrete behaviors; Gifford, 1994; Gifford and Hine, 1994; Muran et al., 1997). The use of the IAS-R by external coders does not assess the display of specific NVBs, but a global impression of overall interpersonal behaviors. Nevertheless, the frequency or duration of several discrete NVB can also be coded. The specific NVB can then be classified in clusters according to the overall control and affiliation dimensions of the ICM, as literature provide evidence for a dimensional conception of the ICM (Lorr and Strack, 1990). To guide which behavior relates to the control or affiliation dimensions of the ICM, one can rely on past research such as Gifford's (1991) mapping of NVB on the ICM, Kiesler and Auerbach's (2003) review of NVBs signaling affiliation and control, or Hall et al.'s (2014) meta-analysis of NVBs related to the vertical dimension of human interactions.

When both the individual's behavior and the interlocutor's inner characteristics have been assessed with measures relating to common overall dimensions, the extent to which one is adapted to the other can be estimated. In that regard, it is of utmost importance to consider that adaptability is not merely defined by similarities or dissimilarities between displayed NVB and inner characteristics of the interlocutor. Measuring similarity and dissimilarity does not tell whether the individuals were displaying their usual pattern of behaviors or actually changing it to fit a particular interlocutor. In order to measure individuals' ability to adapt his or her NVB, one needs to measure their NVB when interacting with at least two different interlocutors. The computation of adaptability scores needs to account for the extent to which the NVB of the individual fit the specific inner characteristics of different interlocutors. An evident measure of correspondence between a behavior variable and an inner characteristics variable across different interactions is a correlation. For example, a study of the effect of physicians' behavioral adaptability to patient preferences used such correlation method (Carrard et al., 2018). Several physicians were videotaped when interacting with four of their patients. Correlations were then computed for each physician between observer ratings of the physicians' behavior in each of their four videotaped interactions and the rating of each of the four corresponding patients' preferences (self-reported before the interaction). The higher the correlation estimate, the more the NVB displayed by a physician in each interaction corresponds to each patient's preferences. Then, the correlation estimates (e.g., Pearson's *r* transformed into Fisher's *z* to avoid added error to the analysis) were used as behavioral adaptability scores predicting interaction outcomes in order to test the beneficial effect of behavioral adaptation to interlocutor's inner characteristics (Carrard et al., 2018).

## Discussion

This perspective paper proposes five main postulations. First, research needs to take a step further than the “one size fits all” approach and thus study adaptation of behaviors, in order to predict better interactional outcomes. Second, the study of non-verbal adaptation is essential, because NVBs highly contribute to the communication between two interactants. Third, behavioral adaptability is more pertinently studied as adaptation to interlocutor's inner characteristics (i.e., expectations or preferences) than to interlocutor's behaviors *per se*. Fourth, the present paper proposes the ICM and its control and affiliation dimensions as the framework to measure both interlocutor's inner characteristics and adapted non-verbal response in a comparable way. Finally, measuring non-verbal adaptability to interlocutors' inner characteristics implies the assessment of different interactions, because it involves a change of NVB according to each interlocutor's specific inner characteristics.

Further research is needed to understand the process of non-verbal adaptation to the interlocutor's inner characteristics, its outcomes, predictors, and covariates. Some evidence suggest that a perceived match between individual's behaviors and interlocutor's expectations or preferences is related to better outcomes such as patient satisfaction (Street et al., 2012), better learning outcomes of students (Young et al., 2003), and more credibility of and attraction to interactional partner (Burgoon and Le Poire, 1993). However, further research on adaptability instead of match are needed, especially for the adaptation of NVB, to confirm its beneficial effect.

Eventually, the ability to display the behaviors that will match the interlocutors' expectations or preferences will depend on one's ability to infer these inner characteristics based on the interlocutor's behaviors. This skill called interpersonal accuracy has been shown to be related to more positive interaction outcomes in sales, clinical interactions, and the workplace (DiMatteo et al., 1986; Byron et al., 2007; Hall et al., 2009). It has been suggested that the relationship between interpersonal accuracy and positive interaction outcome is mediated by behavioral ability (Hall et al., 2016), and a study in patient–physician interactions provides some evidence of this mediation for female physicians (Carrard et al., 2018). Interestingly, a meta-analysis showed that interpersonal accuracy can be efficiently trained with short training sessions (Blanch-Hartigan et al., 2012). Future studies should confirm whether interpersonal accuracy is a predictor of behavioral adaptability, because such training would be an interesting avenue to improve communication competencies. Another predictor of behavioral adaptability is the possession of a large behavioral repertoire and the ability to flexibly change behavioral displays. However, the possibility of training behavioral repertoires or behavioral flexibility is still unknown.

Moreover, future studies should also consider investigating the potential covariates and moderators of non-verbal adaptability. For instance, it has been shown that women are more knowledgeable regarding NVB compared to men (Rosip and Hall, 2004). A meta-analysis further showed that women's interpersonal accuracy is more strongly linked to psychosocial functioning than men's (Hall et al., 2009). Additionally, a study in the physician–patient interaction context showed that the link between physician non-verbal adaptability to patient preferences is linked to more positive patient outcomes for females, but not for males (Carrard et al., 2018). Thus, gender, as well as other potential moderators such as age or culture, should be tested to better understand the non-verbal adaptability process and how it relates to better consultation outcomes.

This perspective paper did not strive to deliver an exhaustive review of the literature on the topic, but to provide some hints for the measure and study of non-verbal adaptation to interlocutor's inner characteristics. My hope is that the limited overview and recommendations presented promotes more research on the topic. Considering that the link between NVB and better interaction outcomes has been acknowledged, the field can move forward with the investigation of non-verbal adaptation to interlocutors' inner characteristics as the foundation of efficient social interactions.

## Data Availability Statement

The original contributions presented in the study are included in the article/supplementary material, further inquiries can be directed to the corresponding author.

## Author Contributions

As sole author, VC, completed all tasks related to the conception and writing of the submitted manuscript.

## Conflict of Interest

The author declares that the research was conducted in the absence of any commercial or financial relationships that could be constructed as a potential conflict of interest.
